# New perspectives: the impact of ketogenic diet on the immune system

**DOI:** 10.1038/s41392-025-02188-w

**Published:** 2025-04-30

**Authors:** Huanzhao Peng, Minyang Fu, Jiong Li

**Affiliations:** 1 Department of Biotherapy, Cancer Center and State Key Laboratory of Biotherapy, West China Hospital, Sichuan University, Chengdu, Sichuan PR China; 2 Department of Food Science and Nutrition, University of Minnesota Twin Cities, Saint Paul, MN USA; 3 Laboratory of Aging Research and Cancer Drug Target, State Key Laboratory of Biotherapy, National Clinical Research Center for Geriatrics, West China Hospital, Sichuan University, Chengdu, Sichuan PR China

**Keywords:** Immunology, Health care

In a recent study published in *Nature Medicine* by Dr. Link, two weeks of dietary intervention affected host immune system significantly.^1^ The major finding was that ketogenic diet significantly promoted pathways and cell enrichment related to the adaptive immune system, with changes in *Actinobacteria*, *Bacteroidetes*, *Firmicutes,* and *Proteobacteria*, where *Firmicutes* being the most affected phylum.

The ketogenic diet is characterized by limited carbohydrate intake, moderate protein intake, and excessive fat intake, which originally was designed for epilepsy treatment, and recently is popularly known for its weight loss functions. A key mechanism underlying its beneficial health outcomes is the induction of ketogenesis, a process that enables the body to utilize lipids as fuel directly.^2^ Subsequently, fatty acid metabolism could be affected by the ketogenic diet. ketogenic diet-mediated fatty acid metabolism, indicated by Dr. Lochner’s study, would impact the regulation of T cell functions.^3^ However, previous studies only focused on individual dietary patterns, whereas Link’s study explored the impacts of both ketogenic and vegan diets.

**Fig. 1:**
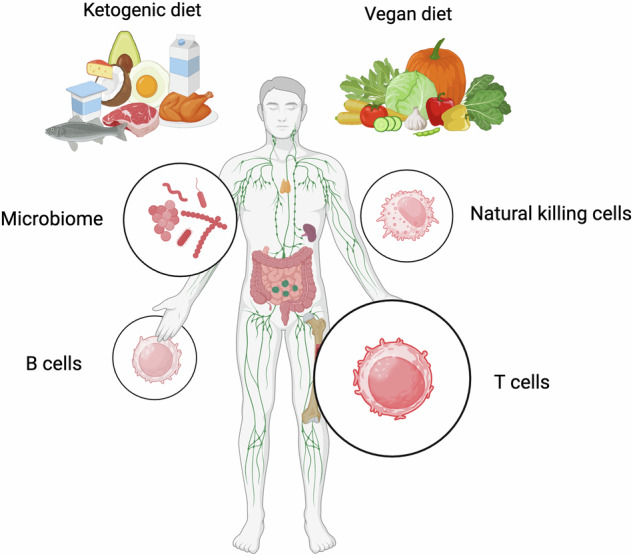
Effect of ketogenic diet and vegan diet on immunity. The ketogenic and vegan diets exert significant effects on immune function through multiple mechanisms. These dietary patterns influence the regulation of various immune cells, including T cells, B cells, and NK cells. Furthermore, they play a crucial role in modulating the gut microbiome, which in turn affects immune responses. Created with BioRender.com

In Link’s study,^1^ both vegan and ketogenic diet caused significant changes in condition of activation and lymphoid composition as shown in Fig. 1. And based on the results of blood transcription module (BTM) and Hallmark analysis, ketogenic diet was observed to promote pathways related to adaptive immunity, such as T cell activation and the enhancement of B cells, plasma cells, and natural killing cells. Similar results were observed in a previous study conducted by Dr. Lochner. Dr. Lochner’s study found out that activation of T cell through mTOR-dependent pathway is associated with the induction of de novo lipid synthesis.^3^ One possible mechanism was the increased oxidative phosphorylation activity by the ketogenic diet, which was related to T cell activation and memory formation. Other studies also demonstrated the association between the ketogenic diet and immunity. In Dr. Lussier’s study, the ketogenic diet intervention resulted in increased CD4 infiltration and consistent T reg numbers.^4^ Furthermore, in Goldberg’s study, the ketogenic diet intervention was found to significantly increase the frequencies and absolute numbers of γδ T cells, and ultimately promoted influenza A virus (IAV) survival through multiple physiological effects.^5^ The following Ingenuity Pathway Analysis (IPA) of disease term confirmed the results from previous BTM and Hallmark analysis, showing increased lymphopoiesis.^1^

Furthermore, Link’s study revealed a highly-interacted, comprehensive network of proteins, metabolites, and microbial enzymes. Principal Components Analysis (PCA) revealed that ketogenic diet had broader influence on the proteosome.^1^ And the following analysis demonstrate a reduction of amino acid metabolism of the microbiome after ketogenic diet consumption, likely resulting from increased dietary amino acids availability and less reliance on microbiome-derived amino acids. Microbiome species were also impacted by the ketogenic diet, as evidenced by changes in microbiome abundance of *Actinobacteria*, *Bacteroidetes*, *Firmicutes* and *Proteobacteria*, *whose metabolites probably affect the immune cells physiological activity*. And Enzyme Commission numbers also showed the pathways related to microbiome abundance were affected with microbial enzymes involved changed in same trend. For example, the biosynthesis of branched-chain amino acids and vitamins B1, B5, and B12 was reduced following ketogenic diet. This was probably due to high dietary intake of amino acids in ketogenic diet. Meanwhile, the ketogenic diet promoted amino acid metabolism and altered lipid metabolism (only saturated fatty acids were increased in patients’ plasma).^1^ Overall, the ketogenic diet significantly impact the patients’ metabolite profile. Taken together, the dataset suggested a significantly interacted network between metabolites, microbiomes, and proteins, which was principally powered by immune factors, amino acids, and lipids.^1^

Link’s study explored the impacts of ketogenic diet and vegan diet intervention on human immunity, metabolites, and microbiota. One of the strengths of this study was its analysis of two diets patterns in one clinical setting, while previous studies mainly focused on one of certain diets. In this way, Link’s study provides an insight into the impact of switching diet patterns on the human immunity and microbiome. And Link’s study highlighted that ketogenic diet promoted adaptive immunity, while the vegan diet upregulated signatures related to innate immunity. With these new findings, we could have a better understanding of how dietary components played a role in human immunity system. This study provided a potential therapeutical strategy and health management to immunity-related physiological conditions via dietary interventions. More further studies are encouraged to focus on this topic and explore the underlying mechanisms.
